# Management of Corneal Ectasia after LASIK with Phototherapeutic Keratectomy Combined with Photorefractive Keratectomy and Collagen Cross-Linking

**DOI:** 10.1155/2019/2707826

**Published:** 2019-02-18

**Authors:** Weiyan Zhou, Hongya Wang, Xiao Zhang, Mingxia Tian, Changxia Cui, Xin Li, Guoying Mu

**Affiliations:** ^1^Department of Ophthalmology, Shandong Provincial Hospital Affiliated to Shandong University, No. 324 Jingwuweiqi Road, Jinan, Shandong, China; ^2^Department of Clinical Laboratory, Shandong Provincial Hospital Affiliated to Shandong University, No. 324 Jingwuweiqi Road, Jinan, Shandong, China; ^3^Department of Ophthalmology, Affiliated Hospital of Jining Medical College, Jining, Shandong, China; ^4^Department of Ophthalmology, Jinan Central Hospital Affiliated to Shandong University, 105 Jiefang Road, Jinan, Shandong 250013, China

## Abstract

**Background:**

To evaluate the efficacy of phototherapeutic keratectomy (PTK) combined with photorefractive keratectomy (PRK) and riboflavin with ultraviolet-A collagen cross-linking (CXL), performed sequentially on the same day, in the management of corneal ectasia after LASIK.

**Methods:**

This retrospective review comprised consecutive patients with corneal ectasia after LASIK. The patients were administered PTK and PRK on the previous corneal flap, and CXL was given on the same day by the same surgeon. The main outcome measures included age, sex, uncorrected distance visual acuity (UDVA), corrected distance visual acuity (CDVA), spherical equivalent refraction, cylinder equivalent refraction, steep and flat keratometries (K), central corneal thickness (CCT), endothelial cell count, corneal haze, and ectasia stability. Mean follow-up period was 6, 12, and 24 months.

**Results:**

Sixteen eyes of twelve patients were included in the study. Twenty-four months after administration of PTK combined with PRK and CXL, a significant improvement in UDVA was observed. Mean cylinder equivalent refraction was significantly reduced at 6, 12, and 24 months postoperatively. However, no significant reduction was observed in spherical equivalent refraction. A significant reduction in the flat K and steep K values was observed. No significant change in mean CCT value was observed. Mean endothelial cell count and morphology were unchanged between preoperative and postoperative patients. In addition, no obvious corneal haze was observed.

**Conclusions:**

PTK combined with PRK and CXL on the same day is a safe and effective treatment in improving visual acuity in selected patients with corneal ectasia after LASIK.

## 1. Introduction

Corneal ectasia after LASIK is characterized as the progressive thinning and steepening of cornea, resulting in refractive aberrations and severe visual loss [1]. All excimer laser procedures can weaken corneal biomechanics because of removing the corneal tissue. Several parameters such as high myopic corrections, thin corneas, and residual corneal thickness <250 *µ*m are considered as major risk factors for corneal ectasia [2].

There are many traditional treatments for corneal ectasia after LASIK, including rigid gas permeable contact lenses and intracorneal ring segments; however, the most frequently used method is corneal transplantation [3]. In recent years, various studies have demonstrated that corneal CXL with no loss in corneal transparency can stiffen the cornea. Several studies have reported the efficacy of corneal CXL as a new treatment for postoperative ectasia [4].

During CXL treatment, the epithelium is removed to permit the absorption of riboflavin solution into the corneal stroma [5]. Excimer laser phototherapeutic keratectomy (PTK) is used to remove the epithelium prior to the refractive surgery [6]. Several combined procedures have been proposed to enhance the efficacy of CXL; however, the most effective method is the combined photorefractive keratectomy (PRK) followed by CXL [7]. Kanellopoulos used transepithelial PTK to remove corneal epithelium before topography-guided PRK and CXL in patients with keratoconus [8]. Studies have shown that PRK can reduce irregular astigmatism and the refractive error, providing patients with improved visual outcomes.

**Figure 1:**
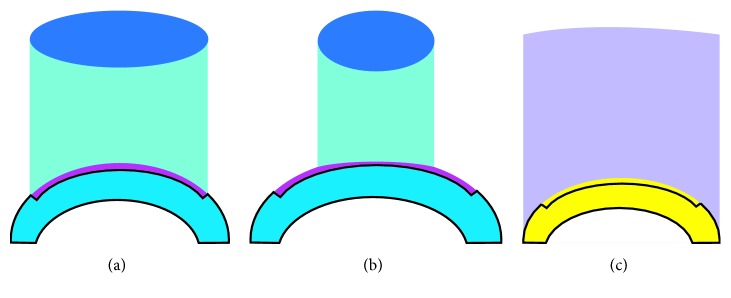
Schematic representation of phototherapeutic keratectomy (PTK) combined with photorefractive keratectomy (PRK) and riboflavin with ultraviolet-A collagen cross-linking (CXL) performed sequentially on the same day in the management of corneal ectasia after LASIK. (a) Removal of the epithelium in the previous corneal flap (PTK). (b) Tissue ablation in the previous corneal flap (PRK). (c) Riboflavin with ultraviolet-A collagen cross-linking (CXL).

The corneal flap is commonly presented in patients with corneal ectasia after LASIK. Randleman et al. reported that the mean measured flap thickness is 138 *µ*m [9]. In our study, the corneal epithelium was removed with PTK. The patients were administered PRK to ablate the corneal tissue in the corneal flap, avoiding the remaining corneal tissue under the flap (Figure 1). The aim of PRK is to normalize the cornea by reducing irregular astigmatism while treating part of the refractive error. Since the corneal tissue of the flap is ablated, we can avoid removal of a significant amount of corneal stroma, thereby maintaining the biomechanical integrity and the thickness of the cornea. However, PTK has not been evaluated in combination with PRK and CXL for the visual enhancement in patients with corneal ectasia after LASIK. The purpose of our study was to evaluate the efficacy of PTK combined with PRK and CXL performed sequentially on the same day in the management of corneal ectasia after LASIK.

## 2. Materials and Methods

### 2.1. Patient Selection

Patients with corneal ectasia after LASIK from the Department of Ophthalmology, Provincial Hospital affiliated to Shandong University, Jinan, China, were included in the study. Once the diagnosis of corneal ectasia after LASIK was confirmed (see the following section), written informed consent was obtained from all the patients. The study was approved by the ethics committee of the Provincial Hospital affiliated to Shandong University, Jinan, China.

A diagnosis of corneal ectasia was carried out for patients with progressive corneal steepening associated with an increasing myopic and/or astigmatic refractive error two or more months after LASIK surgery. The findings were combined with increasing inferior corneal steepening and thinning. Progression of the myopic refractive error with or without progression of the manifest astigmatism, decreasing uncorrected distance visual acuity (UDVA), loss of corrected distance visual acuity (CDVA), progressive inferior corneal steepening on topography, and/or decreasing inferior corneal thickness were observed in all the cases [10].

### 2.2. Clinical Examination

The following evaluations were completed before and after the treatments: age, sex, UDVA, CDVA, refraction, keratometry (K), CCT, endothelial cell count, corneal haze on a scale of 0 to 4 (0 = clear cornea, 1 = mild haze, 2 = moderate haze, 3 = severe haze, and 4 = reticular haze (obstructing iris anatomy)), and ectasia stability as defined by stability in corneal topography [10]. UDVA and CDVA values were recorded in a 20-foot lane using high-contrast Snellen visual acuity testing. Topographical keratometry (K) readings were obtained by corneal topography (ALLEGRO TOPOLYZER VARIO WaveLight Erlangen, Germany). CCT was measured using ultrasound pachymetry (HANDY PACHYMETER SP-100; Nishi-ku, Nagoya 451-0051, Japan). Endothelial cell count was determined in the central cornea by specular microscopy. Slit-lamp microscopy was used to confirm the presence of a LASIK flap and corneal haze.

#### 2.2.1. PTK

PTK was used to remove epithelium in the previous corneal flap. After topical anesthesia with proparacaine eye drops, an excimer laser system with a wavelength of 193 nm (WaveLight EX500 Technologies, Erlangen, Germany) was used for the procedure. PTK ablation was performed using an 8.5 mm zone at intended depth of 50 *µ*m in the previous corneal flap (Figure 1(a)).

#### 2.2.2. PRK

PRK was used to normalize the cornea by reducing irregular astigmatism while treating part of the refractive error. The same surgeon performed all the procedures. PRK was used to ablate the tissue on the previous corneal flap. An excimer laser system with a wavelength of 193 nm (WaveLight EX500 Technologies, Erlangen, Germany) was used for the PRK procedure (Figure 1(b)).

#### 2.2.3. CXL

Immediately after PRK, riboflavin (Medio-Cross riboflavin/dextran solution, 0.1%) was administered topically for 30 min at intervals of 2 mins. Penetration of the cornea and presence of riboflavin in the anterior chamber (riboflavin shielding) were monitored by slit-lamp examination. The cornea was illuminated for 30 min using a UV light lamp (UV-X 1000 system, IROC Innocross AG Co., Switzerland; wavelength 365 nm, irradiance 3 mW/cm^2^, total dose 5.4 J/cm^2^). Riboflavin administration was continued during UV illumination at the same time interval (Figure 1(c)).

After the treatment, a bandage contact lens was applied until the epithelium was completely healed. The patients were examined daily until the epithelium healed and at one, three, and six months postoperatively.

### 2.3. Statistical Analysis

Snellen visual acuities were converted to LogMAR values for analysis. The 2-tailed paired *t*-test was performed. *P* value of <0.05 was considered statistically significant. Statistical analysis was performed using SPSS 18.0 (SPSS, Inc., Chicago, IL).

## 3. Results

Sixteen eyes of twelve patients (8 male and 4 female) with corneal ectasia after LASIK were included in the study. The mean patient age was 28.5 years (range: 12–43 years). Fifteen eyes had a central CCT analyzed by optical pachymetry (350 *µ*m).

All the patients complained of significant pain during the first postoperative night. Most patients' corneal epithelium was healed the fourth day after treatment.

**Figure 2:**
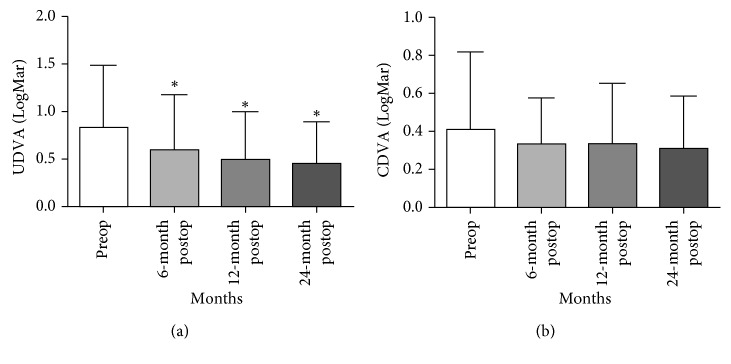
Preoperative and postoperative visual outcomes. (a) Phototherapeutic keratectomy combined with PRK and CXL significantly improved UDVA (*P* < 0.05) at 6, 12, and 24 months follow-up. (b) Phototherapeutic keratectomy combined with PRK and CXL did not significantly improve CDVA at 6, 12, and 24 months follow-up. ^*∗*^Preoperatively vs postoperatively 6, 12, and 24 months *P* < 0.05.

Table 1 shows the preoperative and postoperative visual and refractive outcomes. PTK combined with PRK and CXL significantly improved UDVA (*P* < 0.05) at the 24-month follow-up. Mean UDVA changed from 0.838 ± 0.653 LogMAR preoperatively to 0.594 ± 0.582 LogMAR, 0.494 ± 0.502 LogMAR, and 0.450 ± 0.444 LogMAR at 6, 12, and 24 months postoperatively, respectively (Table 1, Figure 2(a)). A significant improvement in LogMAR UDVA was observed after 24-month postoperative period (*P*=0.002). Mean CDVA improved from 0.413 ± 0.406 LogMAR preoperatively to 0.331 ± 0.61 LogMAR, 0.337 ± 0.318 LogMAR, and 0.313 ± 0.069 LogMAR at 6, 12, and 24 months postoperatively, respectively (Table 1). Improvements in LogMAR CDVA at 6, 12, and 24 months postoperatively were observed; however, the improvements were not significant (*P* > 0.05; Table 1, Figure 2(b)).

**Figure 3:**
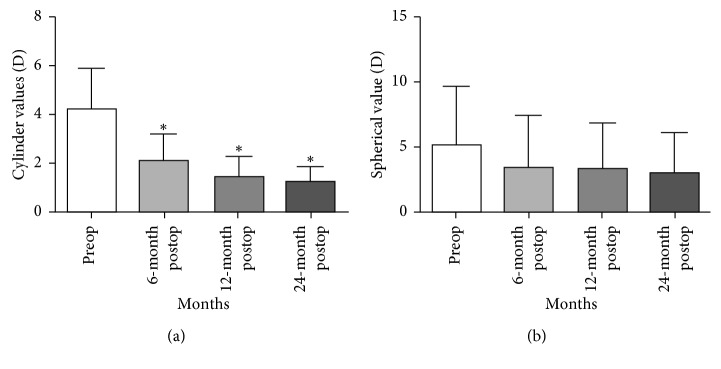
Preoperative and postoperative refractive outcomes. (a) Phototherapeutic keratectomy combined with PRK and CXL significantly reduced cylinder values (*P* < 0.05) at 6, 12, and 24 months follow-up. (b) Phototherapeutic keratectomy combined with PRK and CXL did not significantly reduce spherical equivalent refractions at 6, 12, and 24 months follow-up. ^*∗*^Preoperatively vs postoperatively 6, 12, and 24 months *P* < 0.05.

We detected the preoperative and postoperative patients' refractive status. Mean cylinder equivalent refraction changed from 4.156 ± 1.668 D preoperatively to 2.11 ± 1.099 D, 1.406 ± 0.861 D, and 1.234 ± 0.609 D at 6, 12, and 24 months postoperatively. At the three intervals, all cylinder values were significantly reduced (*P* < 0.05; Table 1, Figure 3(a)). Mean spherical equivalent refraction changed from 5.078 ± 4.55 D preoperatively to 3.391 ± 4.071 D, 3.281 ± 3.55 D, and 2.89 ± 3.145 D at 6, 12, and 24 months postoperatively, respectively. However, at the three intervals, all spherical equivalent refractions were not significantly reduced (*P* > 0.05; Table 1, Figure 3(b)).

**Figure 4:**
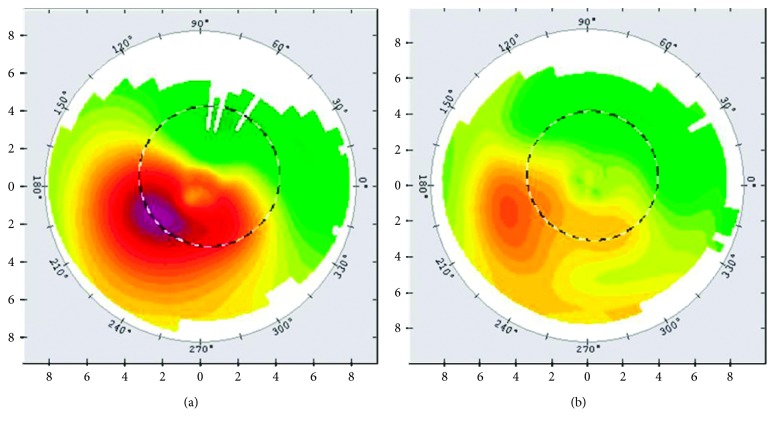
Preoperative and postoperative 24-month follow-up of representative corneal topography. (a) Preoperative corneal topography. (b) Corneal topography follow-up at 24-month after same day PTK, PRK, and CXL treatment in patients with corneal ectasia after LASIK.

**Figure 5:**
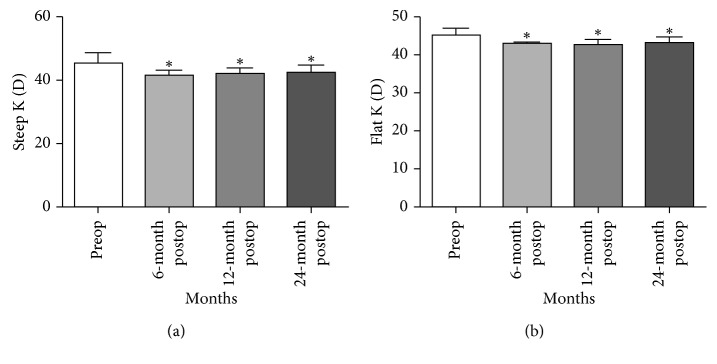
Preoperative and postoperative keratometry. (a) Phototherapeutic keratectomy combined with PRK and CXL significantly reduced the steep K values (*P* < 0.05) at 6, 12, and 24 months follow-up. (b) Phototherapeutic keratectomy combined with PRK and CXL significantly reduced the flat K values at 6, 12, and 24 months follow-up. ^*∗*^Preoperatively vs postoperatively 6, 12, and 24 months *P* < 0.05.

Topographic improvement (Figure 4) and flattening of the cornea were observed, with a significant reduction in the steep K and flat K values (both *P* < 0.05; Table 1). Steep K values changed from 45.675 ± 3.239 D preoperatively to 41.83 ± 1.326 D, 42.312 ± 1.628 D, and 42.612 ± 2.302 D at 6, 12, and 24 months postoperatively. Mean steep keratometry readings improved significantly at postoperative interval (*P* < 0.005; Table 1, Figure 5(a)). Flat K values changed from 45.594 ± 1.696 D preoperatively to 43.125 ± 0.705 D, 42.931 ± 1.345 D, and 43.125 ± 1.795 D at 6, 12, and 24 months postoperatively. Mean flat keratometry readings also changed significantly (*P* < 0.05) at postoperative interval (Table 1, Figure 5(b)).

**Figure 6:**
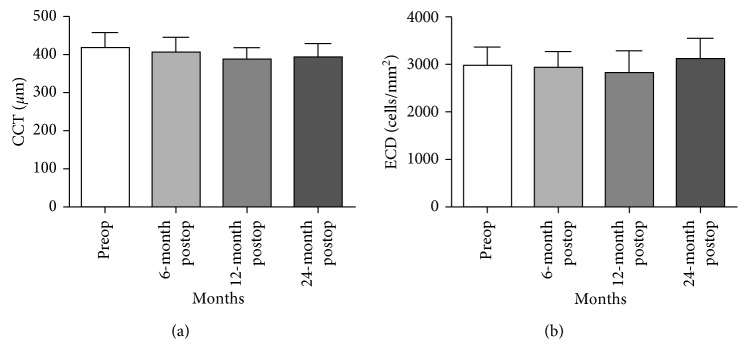
Preoperative and postoperative central corneal thickness and endothelial cell count. (a) Phototherapeutic keratectomy combined with PRK and CXL did not significantly reduce central corneal thickness (*P* > 0.05) at 6, 12, and 24 months follow-up. (b) Phototherapeutic keratectomy combined with PRK and CXL did not significantly reduce the endothelial cell count at 6, 12, and 24 months follow-up.

Mean CCT changed from 416 ± 42.5 *µ*m preoperatively to 406 ± 42.4 *µ*m, 390 ± 30.1 *µ*m, and 393 ± 38.0 *µ*m at 6, 12, and 24 months postoperatively. However, no significant change was observed (*P* > 0.05) at any postoperative interval (Table 1, Figure 6(a)). No change was observed in the mean endothelial cell count and morphology in preoperative and postoperative groups (2979 ± 393 cells/mm^2^ preoperatively and 2936 ± 349 cells/mm^2^, 2823 ± 456 cells/mm^2^, 3110 ± 441 cells/mm^2^ postoperatively) (*P* > 0.05; Table 1, Figure 6(b)).

**Figure 7:**
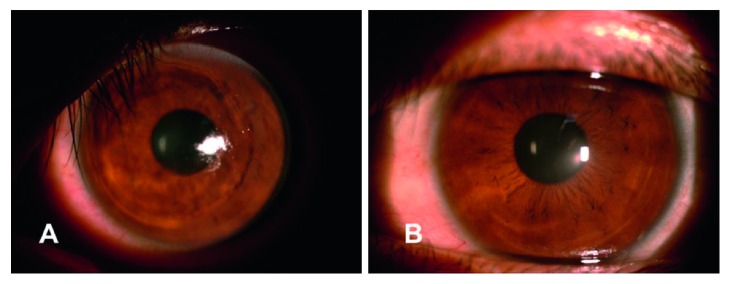
Preoperative and postoperative 24-month follow-up of representative slit-lamp photographs. (A) Representative slit-lamp photographs of patient's cornea treated with PTK and PRK before the CXL. (B) Slit-lamp photographs of the patient's cornea at 24-month follow-up.

Following the treatment, no serious complications related to any of the three treatments (PTK, PRK, or CXL) were reported during the follow-up period. Specifically, no eye developed corneal haze (Figure 7).

## 4. Discussion

Corneal ectasia is characterized by progressive thinning and steepening of the cornea, which can severely impact patients' vision. The most frequent incidence of iatrogenic corneal ectasia is attributed to LASIK [11]. The prevalence of myopia in China is high; many people have undergone surgery of LASIK. Until recently, corneal transplantation has been the most used treatment for corneal ectatic disorders. However, due to the difficult procedure with a long and uncertain visual recovery [12], its use has been limited. Various treatments have been used to postpone or replace the corneal transplantation.

The treatment of corneal ectasia includes two parameters: corneal biomechanical stability and optical improvement of the irregular cornea. The aim of our study was to develop a new technique to treat patients with corneal ectasia after LASIK and improve both corneal stability and functional vision. Reaching functional vision consists of improving UDVA and CDVA and normalization of corneal topography, which indicates that patients are less dependent on contact lenses in order to achieve higher postoperative vision quality. In recent years, many effective tools have been used to treat patients with keratoconus and corneal ectasia. Several combined procedures have also been used to treat corneal ectasia. These methods include intracorneal ring segment implantation (ICRS), phakic intraocular lenses (pIOL), and a phakic toric implantable collamer lens (ICL) combined with CXL. These techniques have different levels of affecting the cornea or anterior chamber structure. But our research can achieve the smallest damage on the eye. Furthermore, most reports of treating patients with corneal ectasia after LASIK had less number of cases and shorter follow-up times.

CXL is a relatively new technique used for the stabilization of ectatic disorders [13, 14]. The technique is based on the absorption of UVA radiation by the cornea after the riboflavin is taken in absorbed by the stroma. This technique can increase the corneal strength and stability by inducing cross-links at the corneal stroma [15]. However, the technique does not increase the visual function of the patient. The need for further refractive correction is required for patients with CXL [16].

PTK uses an excimer laser ablation to remove the epithelium and smoothen the anterior irregular cornea [17, 18]. However, it has been used in combination with CXL for the visual restoration in keratoconus when administered on the same day [19]. PRK is an alternative treatment to improve vision in selected cases of mild keratoconus. The aim of PRK is to remodel the shape of the cornea and decrease irregular astigmatism. It has been demonstrated that patients may benefit substantially even with a relatively small correction due of the corneal thickness limitations [20].

In our study, the epithelium was removed by PTK instead of traditional mechanical or alcohol-led epithelial removal. Through this method, the corneal flap can be preserved completely. After the epithelium removal, the patients with corneal ectasia were administered PRK and CXL on the same day. This is the first reported case where series of patients with corneal ectasia were treated with PTK combined with PRK and CXL performed sequentially on the same day.

The major consideration in the planning of this procedure was the postoperative corneal thickness. The corneal flap includes the corneal epithelium, Bowman layer, and corneal stroma. Randleman et al. evaluated fifty eyes of 29 patients' flap thickness in eyes with corneal ectasia after LASIK. The mean measured flap thickness was 138 *µ*m ± 26 *µ*m (SD) (range: 90–220 *µ*m) [9]. In our current study, the epithelium was removed by PTK. The PTK ablation was performed using an 8.5 mm zone at a depth of 50 *µ*m. A maximum ablation depth of 80 *µ*m was chosen for all cases to achieve a decrease in astigmatism and to avoid removing the corneal tissue beyond the corneal flap that would jeopardize the biomechanical integrity of the cornea. Our results showed that without removing corneal stroma, the vision could be corrected in patients with corneal ectasia.

Our results showed that the treatment was relatively safe, effective, and predictable. Patients treated with simultaneous PTK, PRK, and CXL showed a rapid and significant improvement in UDVA at the 24-month follow-up. Mean cylinder equivalent refraction was significantly reduced at 6, 12, and 24 months postoperatively. However, at three intervals, the spherical equivalent refractions were not significantly reduced. Topographic evaluation of these patients showed marked improvement of irregularity. A significant reduction in the flat K and steep K values was observed. Mean CCT changed from 416 ± 42.5 *µ*m preoperatively to 393 ± 38.0 *µ*m at 24 months postoperatively. However, the change was not significant. Moreover, no significant change was observed in mean endothelial cell count and morphology in preoperative and postoperative groups. The change may be explained by either corneal structural changes, continuous epithelial remodeling, or even the patient's adaptation to the new optical conditions.

## 5. Conclusion

In conclusion, no serious complications or side effects of the treatments were observed in any participant. Simultaneous PTK and PRK treatments followed by CXL seem to be a promising treatment for patients with corneal ectasia. Administering this technique with careful observance of safety could offer patients with corneal ectasia after LASIK an opportunity to gain functional vision, avoid long-term complications such as use of contact lens, and reduce the need for corneal transplantation. Significant improvement in clinical vision and the stability of the ectasia during the postoperative follow-up lead us to believe that the simultaneous approach of PTK, PRK, and CXL may offer an alternative solution to corneal ectasia after LASIK.

## 6. Limitations

However, we should pay attention to the corneal flap thickness that is limited, so this method is not suitable for patients with high astigmatism. In addition, the longer follow-up and larger patient sample are is also required to validate the safety and stability of the combined treatment procedure.

## Figures and Tables

**Table 1:** Preoperative and postoperative patient data for visual acuity, refraction, keratometry, CCT, and endothelial cell density.

	Preop	6 month postop	12 month postop	24 month postop
LogMar UDVA	0.838 ± 0.653	0.594 ± 0.582^*∗*^	0.494 ± 0.502^*∗*^	0.450 ± 0.444^*∗*^
LogMar CDVA	0.413 ± 0.406	0.331 ± 0.61	0.337 ± 0.318	0.313 ± 0.069
Cylinder value (D)	4.156 ± 1.668	2.11 ± 1.099^*∗*^	1.406 ± 0.861^*∗*^	1.234 ± 0.609^*∗*^
Spherical value (D)	5.078 ± 4.55	3.391 ± 4.071	3.281 ± 3.55	2.89 ± 3.145
Steep K (D)	45.675 ± 3.239	41.83 ± 1.326^*∗*^	42.312 ± 1.628^*∗*^	42.612 ± 2.302^*∗*^
Flat K (D)	45.594 ± 1.69	43.125 ± 0.705^*∗*^	42.931 ± 1.345^*∗*^	43.125 ± 1.795^*∗*^
CCT (*µ*m)	416 ± 42.5	406 ± 42.4	390 ± 30.1	393 ± 38.0
ECD	2979 ± 393	2936 ± 349	2823 ± 456	3110 ± 441

LogMar UDVA: uncorrected distance visual acuity in logarithm of the minimum angle of resolution; LogMar CDVA: corrected distance visual acuity in logarithm of the minimum angle of resolution; Steep K (D): mean steep keratometric values in diopters; Flat K (D): mean flat keratometric values in diopters; CCT: central corneal thickness; ECD: endothelial cell density; Postop: postoperatively; Preop: preoperatively. ^*∗*^Preoperatively vs postoperatively 6, 12, and 24 months *P* < 0.05.

## Data Availability

The data used to support the findings of this study have been deposited in the public repository (https://figshare.com/articles/k1_datas/6610646).
